# Targeted integration in human cells through single crossover mediated by ZFN or CRISPR/Cas9

**DOI:** 10.1186/s12896-018-0474-6

**Published:** 2018-10-19

**Authors:** Xiaofeng Liu, Min Wang, Yufeng Qin, Xuan Shi, Peiqing Cong, Yaosheng Chen, Zuyong He

**Affiliations:** State Key Laboratory of Biocontrol, School of Life Sciences, Sun Yat-sen University, Guangzhou, 510006 People’s Republic of China

**Keywords:** Single crossover, ZFN, CRISPR/Cas9, Knock-in, CCR5

## Abstract

**Background:**

Targeted DNA integration is widely used in basic research and commercial applications because it eliminates positional effects on transgene expression. Targeted integration in mammalian cells is generally achieved through a double crossover event between the genome and a linear donor containing two homology arms flanking the gene of interest. However, this strategy is generally less efficient at introducing larger DNA fragments. Using the homology-independent NHEJ mechanism has recently been shown to improve efficiency of integrating larger DNA fragments at targeted sites, but integration through this mechanism is direction-independent. Therefore, developing new methods for direction-dependent integration with improved efficiency is desired.

**Results:**

We generated site-specific double-strand breaks using ZFNs or CRISPR/Cas9 in the human *CCR5* gene and a donor plasmid containing a 1.6-kb fragment homologous to the CCR5 gene in the genome. These DSBs efficiently drove the direction-dependent integration of 6.4-kb plasmids into the genomes of two human cell lines through single-crossover recombination. The integration was direction-dependent and resulted in the duplication of the homology region in the genome, allowing the integration of another copy of the donor plasmid. The CRISPR/Cas9 system tended to disrupt the sgRNA-binding site within the duplicated homology region, preventing the integration of another plasmid donor. In contrast, ZFNs were less likely to completely disrupt their binding sites, allowing the successive integration of additional plasmid donor copies. This could be useful in promoting multi-copy integration for high-level expression of recombinant proteins. Targeted integration through single crossover recombination was highly efficient (frequency: 33%) as revealed by Southern blot analysis of clonal cells. This is more efficient than a previously described NHEJ-based method (0.17–0.45%) that was used to knock in an approximately 5-kb long DNA fragment.

**Conclusion:**

We developed a method for the direction-dependent integration of large DNA fragments through single crossover recombination. We compared and contrasted our method to a previously reported technique for the direction-independent integration of DNA cassettes into the genomes of cultured cells via NHEJ. Our method, due to its directionality and ability to efficiently integrate large fragments, is an attractive strategy for both basic research and industrial application**.**

**Electronic supplementary material:**

The online version of this article (10.1186/s12896-018-0474-6) contains supplementary material, which is available to authorized users.

## Background

Genome editing techniques such as ZFNs (zinc-finger nucleases), TALENs (transcription activator-like effector nucleases), and the CRISPR/Cas9 (clustered regularly interspaced palindromic repeats/CRISPR-associated protein 9) system, have greatly improved the efficiency and accuracy of site-specific modifications in cell lines and organisms. ZFNs use protein-DNA interactions for targeting, while the CRISPR/Cas9 system uses simple RNA-DNA base-pairing rules for targeting [1]. Both ZFNs and CRISPR/Cas9 have been used to efficiently create knock-out alleles in mammalian cells by inducing DNA double-strand breaks (DSBs) which are repaired through the error-prone non-homologous end joining (NHEJ) mechanism. However, knock-in of DNA cassettes at defined loci using ZFNs or CRISPR/Cas9 and homology-directed repair (HDR) is generally less efficient [1], since NHEJ predominates over HDR for DSB repair [2–4]. Knock-in of DNA cassettes into the genomes of mammalian cells is generally achieved through double crossover with a linear donor containing two flanking homology arms and is aided by the presence of genome editors. This method has a relatively lower frequency of integration (10^− 6^–10^− 5^) [5, 6]. In contrast to gene knock-in in mammalian cells, integrating DNA cassettes can be achieved more easily in microorganisms through single “Campbell-like” crossover with a circular plasmid DNA containing a region of homology to a genomic target-locus [7]. Single crossovers allow the integration of multiple copies of expression vectors in yeast [8], and have the advantage of being able to integrate larger DNA fragments into specific genomic loci compared to double crossover [4].

## Results

**Fig. 1:**
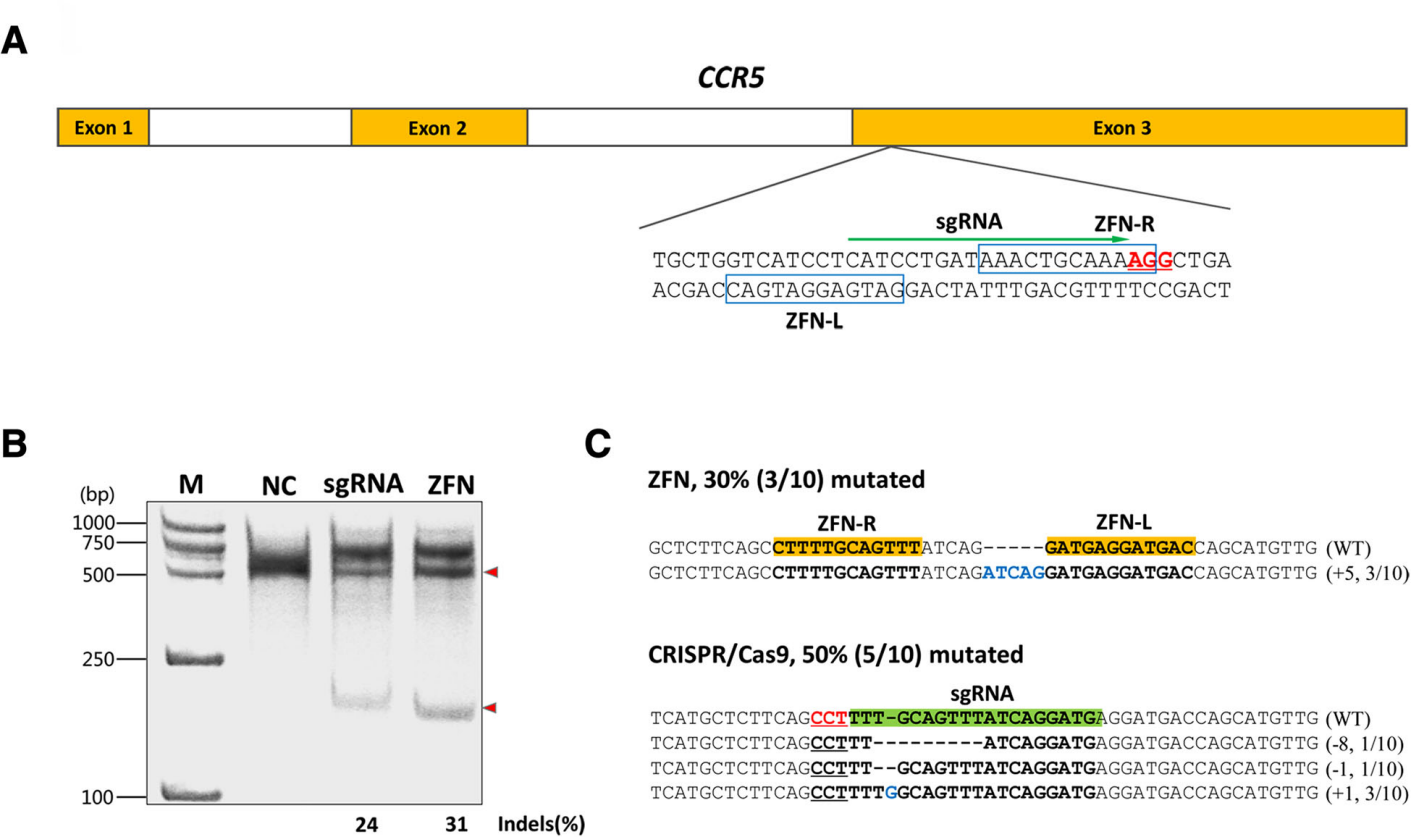
ZFN and sgRNA design and detection of targeted cutting activities. **a** Schematic diagram of the target sites of the designed sgRNA and the ZFN pair in the human *CCR5* gene. The green arrow indicates the sequence used for the guide segment of sgRNA. The NGG nucleotide protospacer adjacent motif (PAM) sequences are shown in red and are underlined. The binding sites of the ZFN pair (ZFN-L and ZFN-R) are marked by blue boxes. **b** The frequency of CRISPR/Cas9- and ZFN-induced mutations as determined by the T7E1 assay. The numbers at the bottom of the gel indicate the mutation percentages estimated based on band intensities measured using ImageJ. NC represents negative control. **c** DNA sequences of the wild-type (WT) and mutant clones. The target sites of the ZFN pair and sgRNA are shown in yellow and green bars, respectively. The PAM sequence is shown in red and underlined. Dashes and blue letters represent deleted and inserted bases, respectively. The number of inserted or deleted bases and the ratio of the number of mutant clones to the number of total clones are indicated in the parentheses. Mutation frequencies were obtained by dividing the total number of mutant clones by the number of total clones

We tested whether targeted integration of multiple copies of a reporter expression cassette can be achieved in human cells through single crossover recombination upon the introduction of double-strand breaks by ZFN or CRISPR/Cas9. As proof of concept, we assembled a pair of ZFNs targeting the human *CCR5* gene based on a previous study [9, 10], and designed an sgRNA targeting the same locus on the human *CCR5* gene (Fig. 1a and Additional file 1: Table S1). To examine the in vivo cleavage activities of the designed ZFNs and CRISPR/Cas9, we transfected HeLa cells with the vector pX330, which co-expresses Cas9 and sgRNA, or with vectors expressing the ZFN pair. Two days after transfection, genomic DNA was prepared for a T7 endonuclease I (T7E1) assay (Additional file 1: Table S2) and TA cloning analysis. The results of the assays show that both sgRNA and ZFN were able to induce NHEJ at their target sites with comparable efficiencies (Fig. 1b and c).

**Fig. 2:**
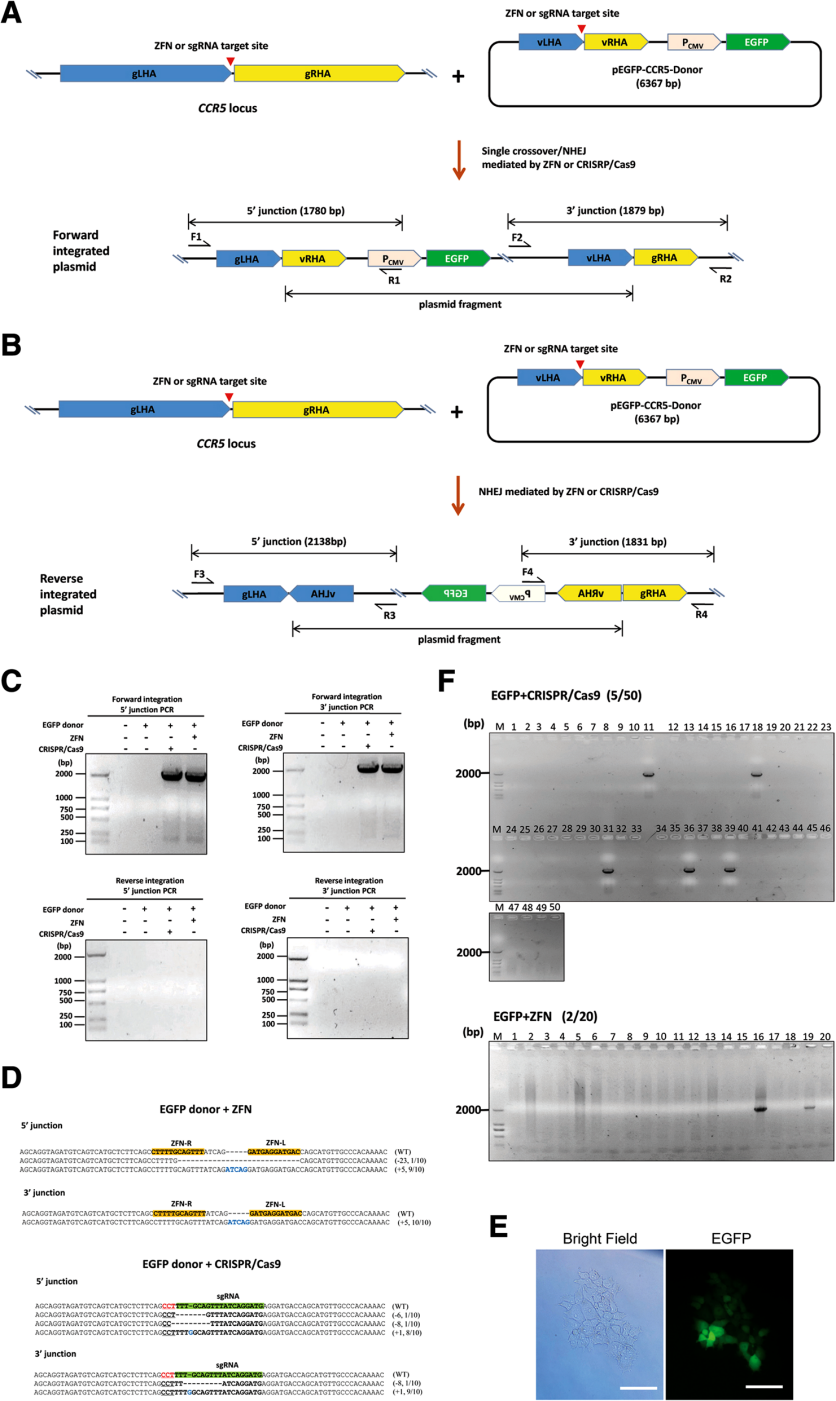
Targeted integration of a single donor plasmid into the *CCR5* locus in HeLa cells through single crossover. **a** Schematic diagram of forward integration of the EGFP donor plasmid into the human genome through the generation of double-strand breaks at the target sites of CRISPR/Cas9 or ZFN in the genome and plasmids via single crossover or NHEJ. Two homology arms flanking the target sites of the engineered nucleases are shown in blue and yellow. vLHA and gLHA represent the left homology arms on the vector and genome, respectively; vRHA and gRHA represent the right homology arms on the vector and genome, respectively. **b** Schematic diagram of reverse integration of the EGFP donor plasmid into the human genome via NHEJ through the generation of double-strand breaks at the target sites of CRISPR/Cas9 or ZFN in the genome and plasmids. **c** Targeted knock-in of donor plasmids in the forward orientation but not in the reverse orientation by ZFN or CRISPR/Cas9 was detected by junction PCR. **d** DNA sequences of the wild-type (WT) and mutant clones. The binding sites of the ZFN pair and sgRNA are shown in yellow and green bars, respectively. The PAM sequence is shown in red and underlined. Deleted and inserted bases are indicated by dashes and blue letters, respectively. The number of inserted or deleted bases and the ratio of the number of mutant clones to the number of total clones are indicated in the parentheses. **e** Brightfield and fluorescence microscopy images of HeLa clonal cells. Scale bar = 50 μm. **f** The frequencies of targeted integration through single crossover mediated by CRISPR/Cas9 or ZFN was detected through junction PCR (represented by the 5′ junction PCR results; similar results of the 3′ junction PCR analysis are not shown)

**Fig. 3:**
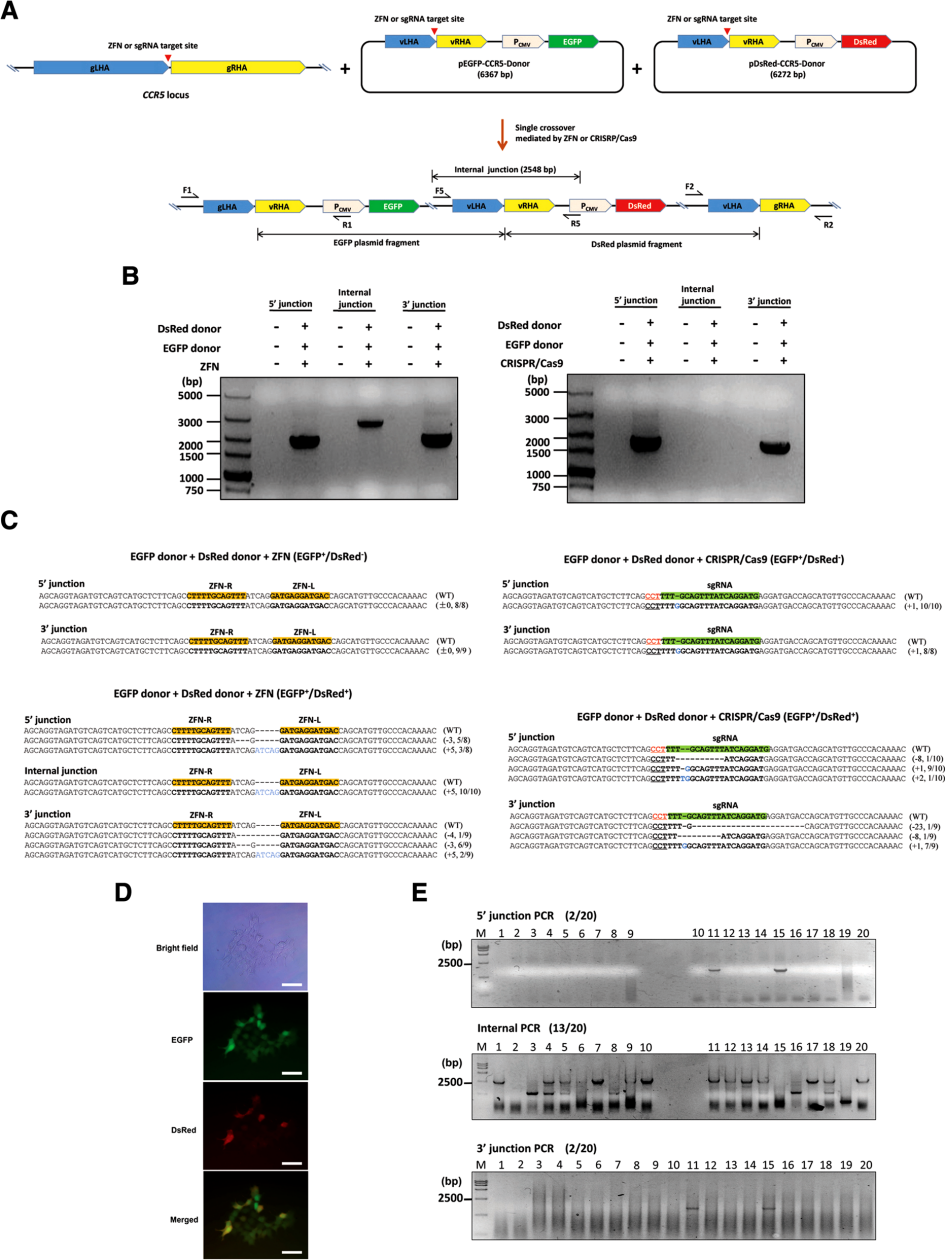
Targeted integration of multiple donor plasmids into the *CCR5* locus in HeLa cells through single crossover. **a** Schematic diagram of the successive integration of EGFP and DsRed donor plasmids into the *CCR5* locus in HeLa cells through single crossover. **b** Junction PCR analysis of targeted integration. **c** Sequence analysis of induced mutations at target sites of the junction PCR amplicon from HeLa cells transfected with only the EGFP donor plasmid (EGFP^+^/DsRed^−^), and cells transfected with both EGFP and DsRed donor plasmids (EGFP^+^/DsRed^+^). The binding sites of the ZFN pair and sgRNA are shown in yellow and green bars, respectively. The PAM sequence is shown in red and underlined. Deleted and inserted bases are presented in dashes and blue letters, respectively. The number of inserted or deleted bases, and the ratio of the number of mutant clones to the number of total clones are indicated in the parentheses. **d** Brightfield and fluorescence microscopy images of clonal HeLa cells. Scale bar = 50 μm. **e** The frequency of targeted multiple integration through single crossover mediated by CRISPR/Cas9 or ZFN was detected through junction PCR analysis

Subsequently, we designed a donor plasmid containing an EGFP reporter and a 1.6-kb fragment homologous to the *CCR5* locus, with the ZFN or CRISPR/Cas9 targeting sites located in the middle (Fig. 2a). We speculated that the in vivo cleavage of a donor plasmid by ZFN or CRISPR/Cas9 could facilitate the integration of the entire plasmid into the *CCR5* locus through a single crossover event at the homology region on the plasmid (Fig. 2a). Alternatively, it is also possible that when the chromosome and the donor plasmid are cut by ZFN or CRISPR/Cas9, the entire plasmid can be integrated into the target site in either forward or reverse orientation through the NHEJ repair pathway, as previously reported [11] (Fig. 2a and b). To test whether our donor plasmid was integrated into the targeted locus through single crossover recombination or NHEJ, HeLa cells stably expressing EGFP were examined through fluorescent imaging and flow cytometry analysis 12 days after transfection. We detected the targeted knock-in of the donor plasmid by ZFN or CRISPR/Cas9 in the forward orientation, but not in the reverse orientation (Fig. 2c) through PCR, using integration site- and donor-specific primers (Fig. 2a and Additional file 1: Table S3). The forward integration of EGFP was replicated in HEK293T cells (Additional file 2: Figure S1A). A previous study has shown that when the target site was cleaved by ZFN in vivo, NHEJ can capture the linearized donor plasmid in both forward and reverse orientations with almost equal frequencies [11]. The failure to detect reverse integration events may indicate the existence of a longer homology region containing nuclease target sites on the donor plasmid, resulting in HDR through single crossover recombination being favored as the main repair pathway. This in turn results in direction-dependent integration and reduces the number of direction-independent integration events mediated by NHEJ. Subsequent analysis of the sequences of the junction between target loci and knocked-in donors in targeted HeLa cells revealed indel events that typically occur after DSB repair by classical NHEJ (Fig. 2d). Interestingly, 90% (9/10 sequences) of the 5′ junction sequences of ZFN-driven knock-ins were found to have an additional spacer inserted in its sequence, and 10% (1/10 sequences) were found to have deletions. On the other hand, 100% of 3′ junction sequences had an additional spacer inserted in its sequence. Among the CRISPR/Cas9-driven knock-ins, 80% (8/10) of 5′ junction sequences were found to have single base insertions, and 20% (2/10) were found to have deletions. On the other hand, 90% (9/10) of 3′ junction sequences had single base insertions and 10% (1/10) had deletions. Similar results were obtained from the analysis of junction sequences in targeted HEK293T cells (Additional file 2: Figure S1B). To evaluate the frequencies of targeted forward integration induced by ZFN or CRISPR/Cas9, we screened for clones derived from single cells stably expressing EGFP by sorting the cells through fluorescence-activated cell sorting (FACS). Compact clonal populations of cells were observed after approximately 9 days of continuous culture (Fig. 2e). The clonal cells were then expanded for an additional 11 days and harvested for junction PCR analysis to detect targeted integration events. Out of 50 clones obtained from CRISPR/Cas9-edited cells, 5 (10%) yielded amplified DNA segments of the expected size (Fig. 2f), while 2 out of 20 clones (10%) obtained from ZFN-edited cells yielded the expected amplicon. Therefore, the knock-in of a 6.4-kb DNA fragment through single crossover recombination in our study is highly efficient (10%). We further investigated whether multiple copies of donor plasmids can be integrated into target sites cut by ZFN or CRISPR/Cas9 through single crossover recombination as is often achieved in yeast. We designed a second donor plasmid based on the first donor plasmid by replacing the EGFP coding sequence with the DsRed coding sequence and keeping the other sequences unchanged (Fig. 3a). The two donor plasmids were separately co-transfected with either ZFN or Cas9/sgRNA expression plasmids into HeLa cells. Twenty days after transfection, cells stably expressing dual fluorescent proteins (Additional file 3: Figure S2A) were collected through FACS and subjected to junction PCR analysis. EGFP-positive and DsRed-negative cells were collected as controls for analysis. In addition to the two pairs of primers used to amplify the 5′ and 3′ junctions, a pair of donor-specific primers were designed to amplify the internal junction in set-ups where multiple donor plasmids were integrated into the target sites (Fig. 3a). The expected amplicons from both 5′ junction and 3′ junction PCR were obtained from both ZFN-driven and CRISPR/Cas9-driven knock-in HeLa cells. However, the expected amplicon from the internal junction was obtained only from ZFN-edited cells (Fig. 3b). The subsequent sequence analysis (Fig. 3c) revealed that both the 5′ and 3′ junction sequences in cells with ZFN-driven knock-in of only the EGFP donor plasmid retained an intact ZFN target site, highlighting the potential for the integration of another donor plasmid. In contrast, both the 5′ and 3′ junction sequences in cells with CRISPR/Cas9-driven knock-in of only the EGFP donor plasmid had single base insertions at the cutting site. This would likely abolish the binding of the sgRNA/Cas9 complex for further cutting and, subsequently, the integration of another donor plasmid. Indels were observed at the 5′, internal, and 3′ junction sequences of ZFN-driven knock-ins of both EGFP and DsRed donor plasmids; more variable indel patterns were observed at both the 5′ and 3′ junction sequences of CRISPR/Cas9-driven knock-ins of both EGFP and DsRed donor plasmids. Similar results were obtained from targeted HEK293T cells (Additional file 3: Figure S2B and C). To evaluate the frequencies of multiply targeted forward integration induced by ZFN, we screened for clones derived from single cells stably expressing both EGFP and DsRed by sorting cells through fluorescence-activated cell sorting (FACS). Compact clonal populations of cells were observed after approximately 9 days of continuous culture (Fig. 3d). The clonal cells were then expanded for an additional 11 days of culture and harvested for PCR analysis to detect multiple targeted integration events. Out of 20 clones obtained from ZFN-edited cells, 2 (10%) yielded 5′ and 3′ junction PCR products with the expected size and 13 clones (65%) yielded the expected internal junction amplicon. Only clone number 11 (5%) yielded all the expected 5′, internal, and 3′ junction PCR products (Fig. 3e).

**Fig. 4:**
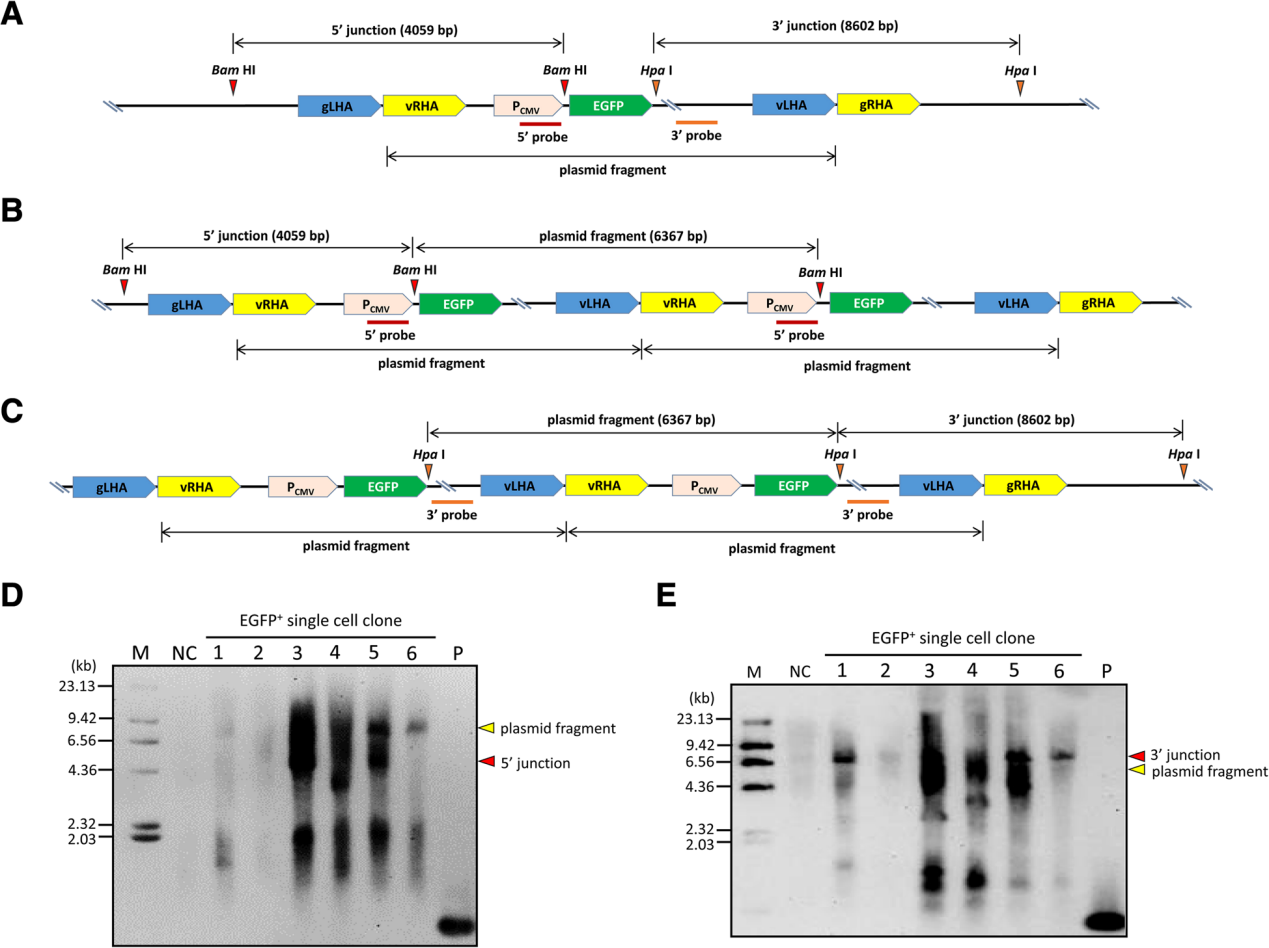
Southern blot analysis of integration events in single cell clones of ZFN-driven knock-in of only the EGFP donor plasmid. **a** The schematic diagram of detecting a 4-kb segment of 5′ junction DNA using *Bam* HI digestion and a 8.6-kb segment of 3′ junction DNA by using *Hpa* I digestion in single copy integration of donor plasmid. The binding positions of the probes (red line for 5′ junction analysis, and orange line for 3′ junction analysis) was indicated. **b** The schematic diagram of detecting a 4-kb segment of 5′ junction DNA and 6.4-kb donor plasmid fragment by using *Bam* HI digestion in multi-copy integration of donor plasmid. Red line indicates the binding site of probe. **c** The schematic diagram of detecting a 8.6-kb segment of 3′ junction DNA and 6.4-kb donor plasmid fragment by using *Hpa* I digestion in multi-copy integration of donor plasmid. Orange line indicates the binding site of probe. **d** Southern blot analysis of the 5′ junction of six single cell clones of ZFN-driven knock-in of only the EGFP donor plasmid. NC represents negative control, where genomic from untransfected cells was used for analysis. P represents positive control, where amplified PCR product using primers for preparing 5′ probe was used for hybridization. **e** Southern blot analysis of the 3′ junction of six single cell clones of ZFN-driven knock-in of only the EGFP donor plasmid. P represents positive control, where amplified PCR product using primers for preparing 3′ probe was used for hybridization

To further characterize the integration events by single crossover, six single cell clones of ZFN-driven knock-in of the EGFP donor plasmid were randomly selected for Southern blot analysis. Genomic DNA isolated from each clone was digested with *Bam* HI and then hybridized with a DIG-labeled probe binding to CMV promoter region to check the 5′ junction of around 4 kb DNA fragment, or digested with *Hpa* I and hybridized with a probe binding to EGFP downstream sequence to check the 3′ junction of 8.6 kb DNA fragment (Fig. 4a). When multi-copy integration occurs, the 6.4 kb plasmid fragment can be released from genomic DNA either by *Bam* HI or *Hpa* I, and detected by hybridization with according probes (Fig. 4b & c). The results showed that junction PCR seemed to underestimate the frequency of targeted integration. Out of the six clones, two (#3 and #5, 33.33%) presented expected size of 5′ junction fragment, and four (#1, #3, #5 and #6, 66.67%) presented expected size of 3′ junction fragment (Fig. 4d & e). Two clones (#3 and #5) presented expected size of both junction fragments, and multi-copy integration, which indicates targeted multi-copy integration through single crossover mediated by ZFN was able to reach to 33.33%. It should be noted that, random integration may happen in addition to targeted multi-copy integration, as blotting signals of DNA fragments with sizes out of the expected sizes of junction fragments was observed in #3 clone (Fig. 4d & e). Incomplete single copy integration at target site seems to happen in #1 clone, as only expected size of 3′ junction fragment was observed, and plasmid fragment and expected size of 5′ junction fragment was not detected (Fig. 4d & e), which implies imperfect recombination could happen at the 5′ junction. Complicated integration of donor plasmid may happen in #6 clone. After *Hpa* I digestion, expected size of 3′ junction fragment was observed, but expected size of 5′ junction fragment and plasmid fragment was not detected by hybridization (Fig. 4e), which implies incomplete integration of single copy of donor plasmid may happen at one allele of *CCR5*. After *Bam* HI digestion, plasmid fragment was detected but expected size of 5′ junction fragment was not detected by hybridization (Fig. 4d). Which presumably suggests incomplete integration of two copies of donor plasmid with one copy breaks between *Bam* HI and *Hpa* I restriction sites flanking the EGFP coding region may happen at another allele of *CCR5* or random site. Thus *Bam* HI digestion was able to release the plasmid fragment (Fig. 4d), however, *Hpa* I was unable to release the plasmid fragment (Fig. 4e). Random integration of single copy of donor plasmid at a region lack of *Bam* HI and *Hpa* I restriction sites may happen in #2 clone, as blotting signal of expected size of both 5′ and 3′ junction fragments, together with the plasmid fragment was not detected (Fig. 4d & e). Random integration of multiple copies of donor plasmid possibly happened in #4 clone, as expected size of both 5′ and 3′ junctions was not detected, but strong blotting signal of plasmid fragment, and DNA fragments with size out of expected size of junction fragments was detected (Fig. 4d & e).

## Discussion

The targeted insertion of a gene of interest into the genome of mammalian cells is a strategy commonly used to overcome positional effects encountered in traditional transgene expression. This can be achieved through the generation of a double-strand break at the target site by engineered nucleases and subsequent HDR via double crossover with a provided plasmid containing a DNA sequence with substantial identity to the region flanking the desired site of integration [3, 10]. However, HDR in human cells is much slower and less efficient than NHEJ [12]. One study showed that in human pluripotent stem cells, the efficiency of knock-in of 3-kb cassettes via CRISPR/Cas9-induced HDR was estimated to be around 10^− 6^–10^− 5^ [6]. Moreover, the efficiency of knock-in through double crossover generally decreases as the size of the insert increases [13]. Therefore, alternative strategies are required to improve the efficiency of targeted insertion of larger DNA fragments into the genome of mammalian cells.

NHEJ-mediated homology-independent targeted integration of in vivo linearized transgene donors has recently been shown to improve targeting efficiency, as demonstrated in one study where site-specific DSBs in the genome and the donor plasmid generated using CRISPR/Cas9 were used to efficiently target ~ 5-kb plasmids into mammalian genomes via NHEJ. They were able to achieve efficiencies of up to 0.17% in HEK293T cells and 0.45% in CHO cells [14]. Our results show that through homology-based single crossover induced by either ZFN or CRISPR/Cas9, the efficiency of the targeted integration of a 6.4-kb plasmid can reach 10% in HeLa cells, which is more efficient than homology-independent targeting via NHEJ. Homology-independent targeted integration via NHEJ generally results in the integration of plasmids in both forward and reverse orientations [11]. In contrast, we showed that targeted integration via homology-based single crossover generally results in the integration of plasmids in the forward orientation, which would be more usefully in applications requiring the uniformity of the orientation of integration. In addition, integration by homology-based single crossover can result in a duplication of the homologous region in the genome, which provides the opportunity to successively integrate multiple copies of the plasmid. This generally increases the expression level dramatically, meeting the requirement for large-scale protein production [15]. Interestingly, we found that CRISPR/Cas9 tended to disrupt the sgRNA-binding sequence in the duplicated homology region, possibly preventing the sgRNA-Cas9 complex from binding the site again for a second round of cutting. Thus, the successive integration of another plasmid donor is inhibited (Fig. 3c and Additional file 3: Figure S2C). In contrast, ZFNs tended to induce small indel mutations in the spacer sequence, but kept the binding sites intact in the duplicated homology region. This would possibly allow ZFNs to bind the site again for another round of cutting, and thus allow the successive integration of additional copies of plasmid donors (Fig. 3b & e; Additional file 3: Figure S2B). This property confers ZFNs an advantage over CRISPR/Cas9 in generating cell lines with stably integrated multiple copies of plasmids. This advantage implies that ZFN should not be totally replaced by CRISPR/Cas9 due to its advantages in specific applications. It is interesting that both the 5′ and 3′ junction sequences of ZFN-driven knock-ins contained insertions of an additional spacer sequence, when co-transfected with only the EGFP donor plasmid (Fig. 2d), while both the 5′ and 3′ junction sequences remained intact in the EGFP-positive/DsRed-negative cells, when co-transfected with both EGFP and DsRed donor plasmids (Fig. 3c). We speculate that the doubled concentration of the donor plasmids used for ZFN-driven knock-in (Fig. 3c), in relative to the concentration of only EGFP donor plasmid used for ZFN-driven knock-in (Fig. 2d), may have contributed to the differences observed in the 5′ and 3′ junctions. As donor plasmids harbor the binding sites of ZFN monomers, a higher number of donor plasmids in the cells would provide more opportunities for ZFN monomer binding. This reduces the chances of the ZFN monomers binding to the genome with the EGFP donor plasmid already integrated for re-cutting, therefore resulting in reduced chances of observing indels at the 5′ and 3′ junction sequences.

It is noticeable that the analysis of multi-copy integration induced by ZFN by junction PCR showed that the internal junction DNA segment could be amplified from 65% of clones, while 5′ and 3′ junction DNA segments can be successfully amplified from only 10% of clones (Fig. 3e). The amplification of the internal junction DNA segment indicates the occurrence of multi-copy integration (Fig. 3a); however, the failure to amplify the 5′ or 3′ junction DNA segments from these clones may imply the occurrence of complex DNA rearrangements (deletions, duplications, insertions, and inversions) at the junctions, possibly induced by illegitimate recombination. Illegitimate recombination is an important competing pathway of homologous recombination for DSB repair in mammalian cells. One study has shown that illegitimate recombination can be stimulated 1,000-fold, as compared to 100-fold for homology recombination at site-specific DSBs [16]. Further characterization of a small portion of clones of ZFN-driven knock-in by Southern blot supports our speculation. Incomplete integration of donor plasmid at target site happened in 33.3% (#1 and #6) clones, as they showed only one putatively perfect junction. Therefore, imperfect recombination could happen at the junctions possibly induced by illegitimate, making junction PCR prone to under estimate the true frequency of targeted integrations through single crossover mediated by ZFN. In addition, we found 50% (#3, #4 and #5) clones presented multi-copy integration, which is comparable to that detected by junction PCR analysis, indicating that junction PCR is able to detect most multi-copy integration events, however, it is hard to discriminate targeted and random integration, as in the three clones presenting multi-copy integration, one (#4 clone) seems to contain random integration.

ZFNs have certain limitations as compared with TALENs. For example, when different integration sites need to be targeted, it is relatively easier to assemble TALEN pairs than ZFN pairs. As TALENs share similar editing patterns with ZFNs, it would be interesting for us to test in the future whether TALENs would induce only small indels in the spacer sequence and keep the binding sites intact after integration of one copy of the donor plasmid into the genome, and subsequently mediate multiple targeted integration through single crossover.

## Conclusion

Compared with direction-independent integration of DNA cassettes into the genomes of cultured cells via the NHEJ repair pathway, the direction-dependent integration of large DNA fragments through single crossover in this study is highly efficient and is capable of mediating multi-copy integration, making it an attractive strategy for both basic research and industrial applications.

## Method

### Vector construction

ZFN pairs targeting the human *CCR5* sequence were assembled based on previous studies [9, 10]. Briefly, the left and right halves of the ZFN coding sequences were synthesized (Generay Biotech, China) and cloned into a ZFN expression vector purchased from Sigma-Aldrich. An sgRNA was designed to target the human CCR5 using the online CRISPR Design Tool (http://crispr.mit.edu/v2). The sequences of the target sites of the designed ZFNs and CRISPR/Cas9 are summarized in Additional file 1: Table S1. For the co-expression of sgRNA and Cas9 in human cells, the synthesized sgRNA oligos were cloned into the pX330 plasmid (Addgene #42230) at the *Bbs* I sites as previously described [17]. To construct the donor plasmid pEGFP-CCR5-Donor, a 1.6-kb fragment homologous to the *CCR5* locus containing a ZFN or CRISPR/Cas9 target site in the middle was synthesized (Generay Biotech, China) and cloned into the pEGFP-N1 vector (Clontech, USA) at the *Ase* I restriction site (Fig. 2a). The donor plasmid pDsRed-CCR5-Donor was generated by replacing the EGFP coding region with the synthesized DsRed-coding DNA segment (Generay Biotech, China).

### Cell culture and transfection

HeLa cells or HEK293T cells were seeded at 1.0 × 10^5^ cells/well in a 24-well plate, cultured with 10% fetal bovine serum-DMEM, and incubated at 37 °C with 5% CO_2_. Upon 70–90% confluence, the cells were transfected with 150 ng each of the plasmids coding for the ZFN pair or 150 ng of the Cas9/sgRNA co-expression vector pX330 using Lipofectamine 3000 (Thermo Fisher Scientific). At day 3 post-transfection, genomic DNA was isolated from cells using the DNeasy Blood & Tissue Kit (Qiagen) following the manufacturer’s protocol and was subsequently analyzed through PCR and the T7E1 assay. For targeted integration using donor plasmids, 150 ng each of the plasmids encoding the ZFN pair, 100 ng of pEGFP-CCR5-Donor, and 100 ng of pDsRed-CCR5-Donor were transfected into the cells. For CRISPR/Cas9-mediated knock-in, 150 ng of the Cas9/sgRNA co-expression vector pX330, 100 ng of pEGFP-CCR5-Donor, and 100 ng of pDsRed-CCR5-Donor were transfected into the cells.

### T7E1 assay

The T7E1 assay was performed as previously described [18]. Briefly, primers (Additional file 1: Table S2) were designed to amplify a 750-bp fragment across the target sites of the designed ZFNs or sgRNA from genomic DNA of cells transfected with ZFN or CRISPR/Cas9. The PCR products were denatured and annealed to form heteroduplex DNA, which could be cut by treatment with 0.5 μL T7E1 (NEB, USA) for 30 min at 37 °C. The digestion products were run on a 10% polyacrylamide Tris-borate-EDTA (TBE) gel. After staining the gel with SYBR Gold, mutation frequencies were calculated based on relative band intensities determined using the software Image J [17].

### Sequencing analysis

The *CCR5* gene fragment containing the ZFN and sgRNA target sites were amplified using LA Taq DNA polymerase (Takara, Japan) and the designed primers (Additional file 1: Table S2). PCR products were analyzed by agarose gel electrophoresis, purified using a gel extraction kit (OMEGA, USA), and then cloned into the vector pMD18-T (Takara, Japan). Cloned plasmids were sequenced using M13 primers. Similarly, junction PCR products (Additional file 1: Table S2) were cloned into pMD18-T vector for Sanger sequencing.

### Clonal cell culture

For clonal expansion of single cells stably expressing EGFP or co-expressing EGFP and DsRed, a single cell from the cell population edited by engineered nucleases and donor plasmids was seeded into each well in a 96-well plate after fluorescence-activated cell sorting (FACS). The cells were cultured in condition medium (medium from log-phase HeLa cells filtered through a 0.45 μm pore size filter, and supplemented with 10% fetal bovine serum (FBS)) for 9 days to form a compact clonal population of cells. The clonal cells were then cultured for an additional 11 days and harvested for junction PCR analysis to detect targeted integration events.

### Southern blot assay

Genomic DNAs were isolated from single cell clones of ZFN-driven knock-in of only the EGFP donor plasmid for Southern blot analysis. 8 μg of isolated DNA was digested with *Bam* HI for 5′ junction analysis, or *Hpa* I for 3′ junction analysis. Digested samples were separated on a 0.7% agarose gels at 25 V overnight. DNA in 2 × standard saline citrate (SSC) was transferred to a charged nylon membrane (Roche). Probes for detecting donor plasmids integration were prepared as followings: probes were PCR-amplified from donor plasmid using primers, CCR5-5F and CCR5-5R for 5′ junction analysis and CCR5-3F and CCR5-3R for 3′ junction analysis (Additional file 1: Table S4). DNA probes were labelled by PCR amplification in the presence of digoxigenin-11-dUTP according to the instruction of PCR DIG Probe Synthesis Kit (Roche). For Southern blot hybridizations, nylon membranes were prehybridized for 2 h at 37 °C in hybridization solution without labelled probe and then hybridized separately at 37 °C with specific DNA probes overnight. The DNA was washed twice in 2 × SSC and 0.1% SDS (15 min each time) at room temperature, and once in 1× SSC and 0.1% SDS for 15 min at 65 °C, and was blocked in blocking buffer for 30 min. DNA hybridizations and detection were conducted by using the DIG labelling and CSPD substrate according to the instruction of DIG High Prime DNA Labling and Detection Starter II (Roche), and were exposed on an X-ray film.

## Additional files

Additional file 1: **Table S1.** Target sequences within the *CCR5* gene of engineered nucleases. **Table S2.** Primers used for T7E1 assay. **Table S3.** Primers used for junction PCR analysis. **Table S4.** Primers used for Southern blot assay. (DOCX 16 kb)

Additional file 2: **Figure S1.** Targeted integration of single donor plasmid at the *CCR5* locus in HEK293T cells through single crossover. (JPG 1217 kb)

Additional file 3: **Figure S2.** Targeted integration of multiple donor plasmids at the *CCR5* locus in HEK293T cells through single crossover. (JPG 2027 kb)
