# Supplementary material for: Targeted integration in human cells through single crossover mediated by ZFN or CRISPR/Cas9
Source: BMC Biotechnol. 2018 Oct 19;18:66. doi: 10.1186/s12896-018-0474-6 (PMC6194632; doi:10.1186/s12896-018-0474-6)
Supplement: Supplementary file 1 — Table S1. Target sequences within the CCR5 gene of engineered nucleases. Table S2. Primers used for T7E1 assay. Table S3. Primers used for junction PCR analysis. Table S4. Primers used for Southern blot assay. (DOCX 16 kb) [file 12896_2018_474_MOESM1_ESM.docx]

Supplementary table 1. Target sequences of engineered nucleases on human *CCR5* gene.

| Name | Direction | Sequence (5’ to 3’) |
| --- | --- | --- |
| ZFN target site | FP | GTCATCCTCATCCTGATAAACTGCAAAAG |
| CRISPR/Cas9 target site | FP | CATCCTGATAAACTGCAAA |

Supplementary table 2. Primers used for T7E1 assay.

| Name | Sequence (5’ to 3’) | Product size (bp) |
| --- | --- | --- |
| CCR5-1F | GACCCTGTCTCACAACAA | 746 |
| CCR5-1R | GGATGATGAAGAAGATTCC |  |

Supplementary table 3. Primers used for junction PCR analysis.

| Name | Sequence (5’ to 3’) | Product size (bp) |
| --- | --- | --- |
| Forward Integration-1F(L) | ACAGTGATTGGCATCCAG | 1780 |
| Forward Integration-1R(L) | GGCTATGAACTAATGACCC |  |
| Forward Integration-2F(R) | GGGAAACGCCTGGTATCTT | 1879 |
| Forward Integration-2R(R) | GGAGCCTCTTGCTGGAAA |  |
| Reverse Integration-3F(L) | TGAATGGGTATGATGCTT | 2138 |
| Reverse Integration-3R(L) | CTGGATGTGGCTTGACTC |  |
| Reverse Integration-4F(R) | AGGGATAACCGCAATGAT | 1831 |
| Reverse Integration-4R(R) | CCCAGTGGATCGGGTGTA |  |
| Double donors Integration-5F | ATACCTCGCTCTGCTAATCC | 2548 |
| Double donors Integration-5R | TGAGTCAAACCGCTATCCAC |  |

Supplementary table 4. Primers used for Southern blot assay.

| Name | Sequence (5’ to 3’) | Product size (bp) |
| --- | --- | --- |
| CCR5-5F | TGTTCCCATAGTAACGCCAATA | 373 |
| CCR5-5R | GGCGGAGTTGTTACGACATTTT |  |
| CCR5-3F | AGACCGAGATAGGGTTGAGTGT | 428 |
| CCR5-3R | CATGAGCGGATACATATTTGAA |  |
