# Supplementary figures and images for: Targeted integration in human cells through single crossover mediated by ZFN or CRISPR/Cas9
Source: BMC Biotechnol. 2018 Oct 19;18:66. doi: 10.1186/s12896-018-0474-6 (PMC6194632; doi:10.1186/s12896-018-0474-6)

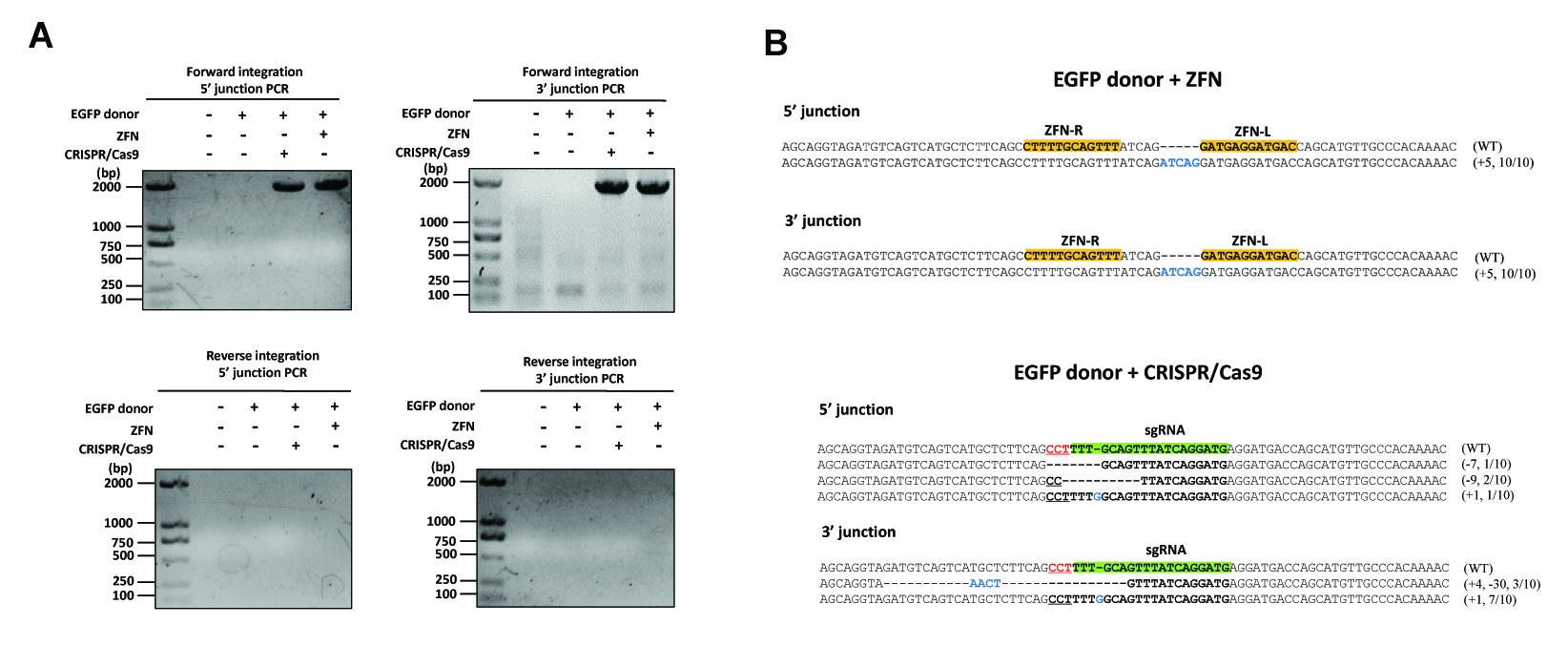

Supplement: Supplementary file 2 — Figure S1. Targeted integration of single donor plasmid at the CCR5 locus in HEK293T cells through single crossover. (JPG 1217 kb) [file 12896_2018_474_MOESM2_ESM.jpg]

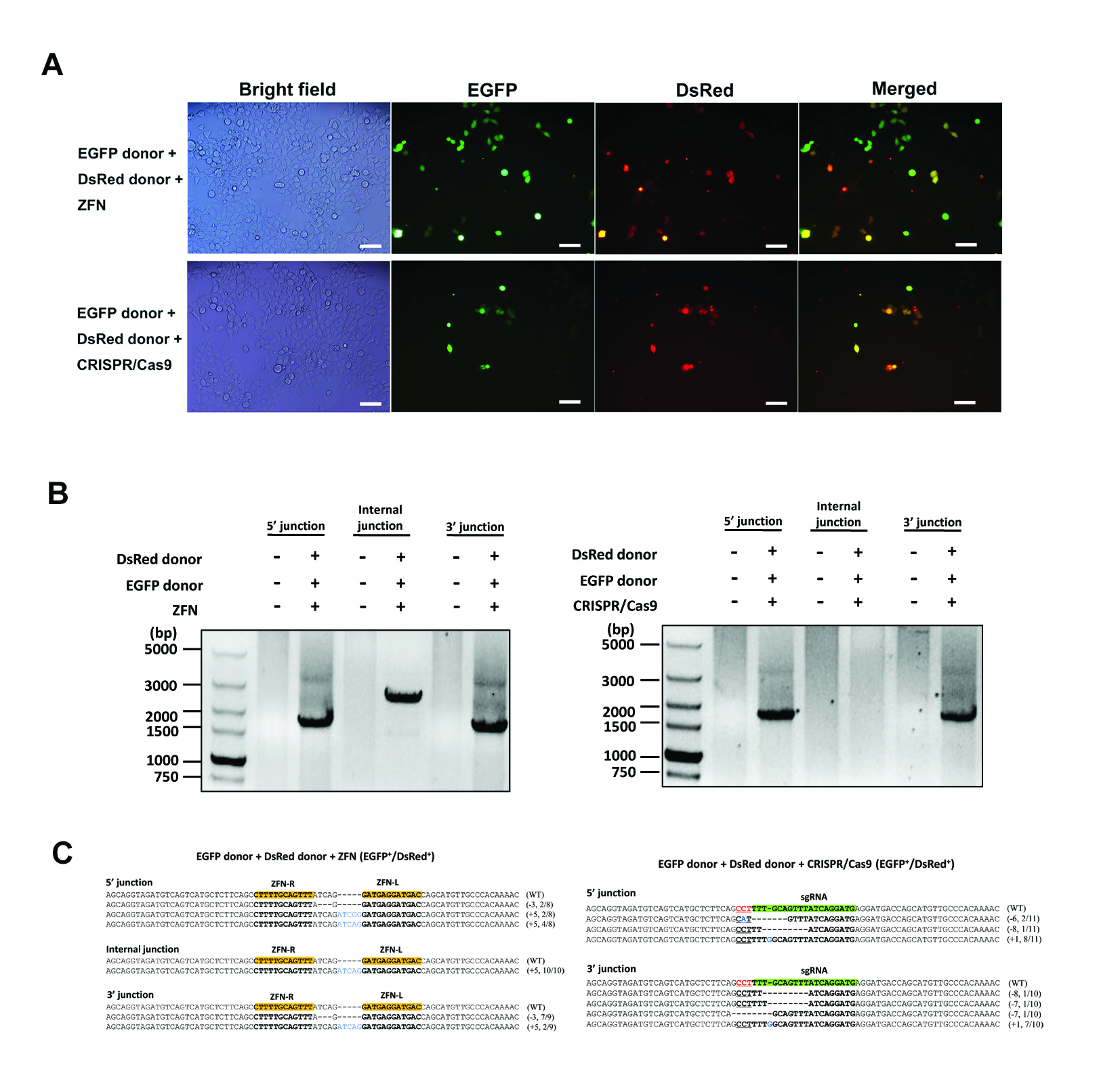

Supplement: Supplementary file 3 — Figure S2. Targeted integration of multiple donor plasmids at the CCR5 locus in HEK293T cells through single crossover. (JPG 2027 kb) [file 12896_2018_474_MOESM3_ESM.jpg]
